# Supplementary material for: Prevalence of SARS-CoV-2 and co-infection with malaria during the first wave of the pandemic (the Burkina Faso case)
Source: Front Public Health. 2022 Dec 12;10:1048404. doi: 10.3389/fpubh.2022.1048404 (PMC9791192; doi:10.3389/fpubh.2022.1048404)
Supplement: Supplementary file 1 [file Data_Sheet_1.docx]

Supplementary Material

# Supplementary Figures and Tables


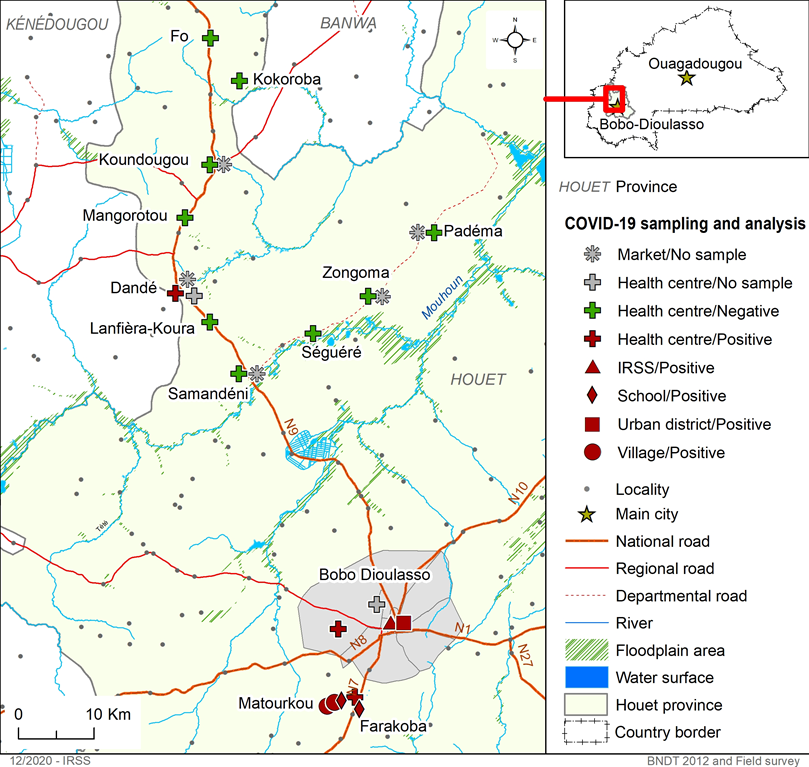


**Supplementary Figure S1.** Map of sampling area in Burkina Faso.


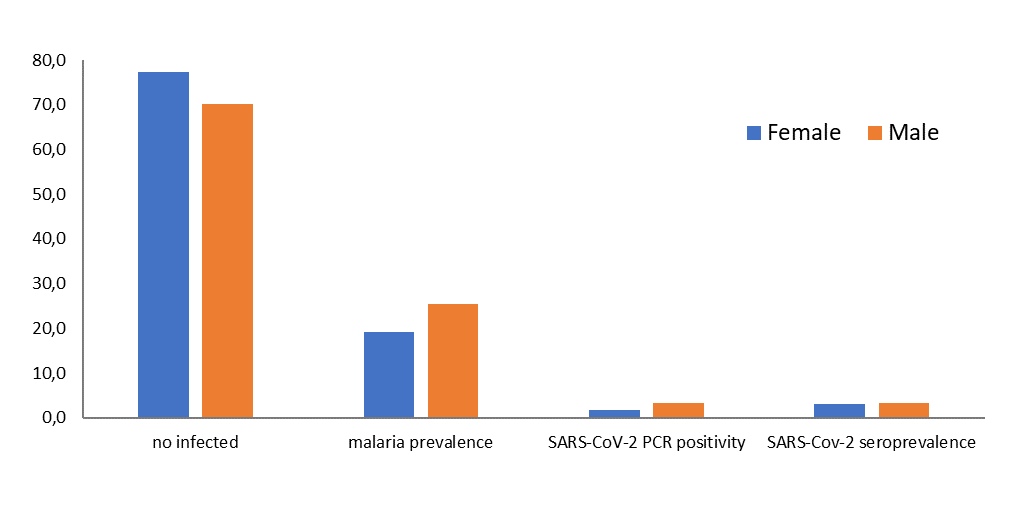


**Supplementary figure S2**. Prevalence of SARS-CoV-2 and malaria positive cases by gender.


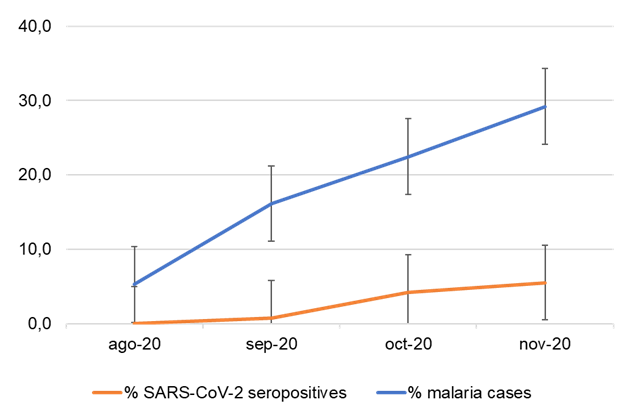


**Supplementary figure S3.** Prevalence of SARS-CoV-2 and malaria over the study period.

**Supplementary table S1.** Representative anti-SARS-CoV-2 seroprevalence studies in Africa. Peer reviewed articles and pre-prints of studies that reported anti-SARS-CoV-2 seroprevalence of general or specific populations in Africa. 32 studies.

| **Subregion** | **Study location** | **Period** | **Study design** | **Participants** | **Sample size** | **Overall seroprevalence** | **Ref.** |
| --- | --- | --- | --- | --- | --- | --- | --- |
| Western Africa | Niger, Nigeria | June 2020 | Cross-sectional | General population | 185 | 25.4% | (1) |
| Western Africa | Calabar, Nigeria | June 2020 | Random | Clinical staff and patients | 66 | 7.6% | (2) |
| Western Africa | Nigeria | August 2020 | Cross sectional non-probability sampling | Blood donors | 113 | 42 % (95% CI: 34–52) | (3) |
| Western Africa | Ibadan, Nigeria | Not indicated | Hospital-based cross-sectional | Health workers | 133 | 45.1% | (4) |
| Western Africa | Enugu, Gombe, Lagos and Nasarawa, Nigeria | October 2020 | Cross-sectional | Households | 10.629 | 25.2% (95% CI 21.8–28.6) Enugu, 9.3% (95% CI 7.0–11.5) Gombe, 23.3% (95% CI 20.5–26.4) Lagos, 18% (95% CI 14.4–21.6) Nasarawa. | (5) |
| Western Africa | Guinea Bissau | November 2020 | Cross-sectional in a cohort. | Health workers | 140 | 18% | (6) |
| Western Africa | Senegal | October-November 2020 | Cross-sectional | Households | 1.463 | 28.4% (95% CI: 26.1-30.8) | (7) |
| Western Africa | Lome, Togo | April-May 2020 | Cross-sectional among high-risk sectors | Healthcare, air transport, police, drivers. | 955 | 0.9% (95% CI: 0.4–1.8) | (8) |
| Western Africa | Ivory Coast | July-October 2020 | Volunteering testing | Gold miners | 1.687 | 25.1% | (9) |
| Western Africa | Sierra Leone | March 2021 | Cross-sectional | Households | 1.893 | 2.6% (95% CI 1.9- 3.4) | (10) |
| Western Africa | Mali | July 2020- January 2021 | Prospective cohort | General population | 2.533 | 58.5% (95% CI: 47.5 to 69.4) | (11) |
| Eastern Africa | Yuba, South Sudan | August-September 2020 | Cross-sectional | Residents | 2.214 | 38.3% (95% CI 31.8–46.5) | (12) |
| Eastern Africa | Addis Ababa, Ethiopia | May 2020 | Convenience | General population | 99 | 3.0%, (95% CI: 0.6–8.6) | (13) |
| Eastern Africa | Dire Dawa, Ethiopia | June-July 2020 | Cross-sectional | Adults | 684 | 3.2% (95 % CI 2.0–4.8) | (14) |
| Eastern Africa | Ethiopia | July 2020 | Cross-sectional | General population | 16.932 | 3.5% (95% CI: 3.2-3.8) | (15) |
| Eastern Africa | Addis Ababa and Jimma, Ethiopia | July-September 2020 | Population-based household | General population | 1.856 | 3.5% (95% CI 1.7-5.4%) Addis Ababa. 1.6% (95%CI 0-4.1%) Jimma. | (16) |
| Eastern Africa | Addis Ababa, Ethiopia | April 2020 | Cross-sectional | General population | 301 | 8.8% (95% CI 5.5-11.6) | (17) |
| Eastern Africa | Kenia | April-June 2020 | National census | Blood donors | 3.098 | 5.6% (95% CI 4.8-6.5) | (18) |
| Eastern Africa | Nairobi, Kenia | November 2020 | Cross-sectional | Residents | 1.164 | 34.7% (95% CI 31.8-37.6) | (19) |
| Eastern Africa | Blantyre, Malawi | May-June 2020 | Longitudinal | Health workers | 500 | 12.3% (CI 8.2 - 16.5) | (20) |
| Central Africa | Yaounde, Cameroon | October-November 2020 | Cross-sectional | Residents | 971 | 29.2% (95% CI 24.3-34.1) | (21) |
| Central Africa | Gabon | July-October 2020 | Prospective | General population | 1.492 | 36.2% | (22) |
| Central Africa | Brazzaville, Republic of  Congo | April-July 2020 | Cross-sectional | General population | 754 | 19.8% | (23) |
| Central Africa | Bukavu, Democratic Republic of Congo | July-August 2020 | Cross-sectional in a cohort. | Healthcare workers | 359 | 41.2% | (24) |
| Northern Africa | Alzintan, Libya | April-May 2020 | Random sampling | Community and healthcare workers | 219 | 2.74% | (25) |
| Northern Africa | Egypt | April-October 2020 | Household cohort | General population | 1.598 | 34.8% | (26) |
| Southern Africa | Zambia | July 2020 | Cross-sectional cluster-survey | Residents | 4.258 | 2.1% (1.1–3.1) | (27) |
| Southern Africa | Zambia | July 2020 | cross-sectional of patients | Outpatients | 1.657 | 8.2% (95% CI 5.1–11.4). | (28) |
| Southern Africa | Cape Town, South Africa | August-September 2020 | Convenience sampling | Workers | 405 | 23.7% | (29) |
| Southern Africa | Eastern Cape, Northern Cape, Free State and KwaZulu Natal, South Africa | January 2021 | Convenience | Blood donors | 4.858 | 62.5% (58.9–66.1) Eastern Cape, 31.8% (25.3-38.3) Northern Cape, 45.5% (39.9-51.1) Free State, 52.1% (49.1-55.2) KwaZulu Natal | (30) |
| Southern Africa | South Africa | November 2020 – April 2021 | Cross-sectional household | HIV-Infected and Uninfected Persons | 7959 | 45.2% (95% CI 43.7- 46.7) | (31) |
| Southern Africa | South Africa | January-May 2021 | Convenience | Blood donors | 16.762 | 47.4% (95% CI 46.2-48.6) | (32) |

**Supplementary table S2.** Detection of samples extracted in pools using the Direct SARS-CoV-2 Realtime PCR Kit (Vircell S.L)

| **Sample alias** | **N gene** | **E gene** | **hRNAse** |
| --- | --- | --- | --- |
| BOB108 | 28.62 | 28.78 | 23.74 |
| BOB108 Pool 1/5 | 31.33 | 31.38 | 26.94 |
| BOB108 Pool 1/10 | 31.86 | 31.92 | 27.94 |
| BOB245 | 34.26 | 34.29 | 25.78 |
| BOB245 Pool 1/5 | 36.56 | 36.99 | 27.45 |
| BOB245 Pool 1/10 | 38.57 | 38.91 | 27.55 |
| Positive control | 29.75 | 28.50 | 28.96 |
| Negative control | NA | NA | NA |

**Supplementary table S3.** Description of lineages found at Burkina Faso in 2020 (https://cov-lineages.org/lineage_list.html)

| **Lineage** | **Most common countries** | **Earliest date** | **Description** |
| --- | --- | --- | --- |
| A.19 | Cote_d'Ivoire 49.0%, Burkina_Faso 40.0%, Italy 3.0%, Australia 2.0%, France 1.0%, | 2020-06-04 | Cote d'Ivoire/ Burkina Faso lineage |
| A.21 | France 26.0%, Burkina_Faso 25.0%, USA 11.0%, Portugal 4.0%, Gabon 4.0% | 2020-04-10 | Mali/ Burkina Faso lineage |
| B.1.1.404 | Burkina_Faso 62.0%, Germany 15.0%, Ghana 6.0%, Italy 6.0%, Togo 3.0% | 2020-05-25 | Ghana, Burkina Faso, Luxembourg, Germany |
| B.1.1.118 | USA 98.0%, Germany 1.0%, Canada 1.0% | 2020-04-10 | US lineage (TX) |
| B.1 | USA 45.0%, Turkey 12.0%, United Kingdom 7.0%, Canada 4.0%, France 3.0% | 2020-01-10 | A large European lineage the origin of which roughly corresponds to the Northern Italian outbreak early in 2020. |

**Supplementary table S4.** SARS-CoV-2 and malaria co-infection published studies

| **Ref.** | **Study location (date)** | **Study design** | **Participants (n)** | **COVID-19 (n)** | **Malaria coinfection (n)** | **Malaria Prevalence among COVID-19** | **Overall co-infection prevalence** | **Clinical data** |
| --- | --- | --- | --- | --- | --- | --- | --- | --- |
| (33) | Lagos, Nigeria, (April-May 2020) | Cross-sectional study. | Patients suspected with COVID-19 (617) | 121 | 2 | 1.66% | 0.32% | Asymptomatic (52%), mild (48%) |
| (34) | Dutse, Nigeria (March-July 2020) | Cross-sectional study | Participants (74): patients with COVID-19 (54), healthy controls (20) | 54 | 34 | 62.9% | 45.9% | NS |
| (35) | Rivers State, Nigeria (2020) | Cross-sectional study | Patients with COVID-19 (300) | 300 | 300 | 100% | 100% | NS |
| (36) | Kinshasa, Democratic Republic of Congo | Retrospective cohort study | Patients with COVID-19 (160) | 160 | 1 | 0.63% | 0.63% | Mild (57%), moderate (12%), severe (31%) |
| (37) | Uganda (April-October 2020) | Exploratory prospective | Patients with COVID-19 (597) | 597 | 70 | 12% | 12% | Asymptomatic (43%), mild (39%), moderate (8%), severe (8%), critical (3%) |
| (38) | Uganda (March –December 2020) | Prospective Cohort Study | Patients with COVID-19 (270) | 270 | 4 | 1.5% | 1.5% | Symptomatic, mildly symptomatic or asymptomatic |
| (39) | Malawi (April-September 2020) | Prospective Cohort Study | Patients suspected with COVID-19 (87) | 66 (41 PCR+, 25 IgG+/PCR-) | 3 | 4.5% | 3.45% | Severe acute respiratory infection (SARI) |
| (40) | Mumbai, India (April-October 2020) | Retrospective cohort study | Front-line health-care workers (3.711) | 491 | 27 | 5.5% | 0.73% | Mild (73.9%), moderate (12.5%), severe (2.4%) |

NS: not specified

**Supplementary table S5.** SARS-CoV-2 positive cases analysed by *Plasmodium falciparum* sensitive PCR targeting a region of the mitochondrial *cox1* gene.

| **Sample Alias** | **Age (years)** | **Group Age** | **Gender** | **COVID-19 Ig** | **SARS-Cov-2 PCR** | **Malaria microscopy** | ***P. falciparum* PCR** |
| --- | --- | --- | --- | --- | --- | --- | --- |
| BOB20 | 15 | B. 13-20 | M | negative | **positive** | negative | negative |
| BOB43 | 43 | D. >40 | F | **positive** | **positive** | negative | negative |
| BOB49 | 53 | D. >40 | M | **positive** | **positive** | negative | negative |
| BOB86 | 38 | C. 21-40 | F | negative | **positive** | negative | negative |
| BOB108 | 71 | D. >40 | M | **positive** | **positive** | negative | negative |
| BOB113 | 56 | D. >40 | M | negative | **positive** | negative | negative |
| BOB153 | 69 | D. >40 | F | **positive** | **positive** | negative | negative |
| BOB155 | 50 | D. >40 | M | negative | **positive** | negative | negative |
| BOB156 | 53 | D. >40 | M | negative | **positive** | negative | negative |
| BOB159 | 41 | D. >40 | F | negative | **positive** | negative | negative |
| BOB192 | 25 | C. 21-40 | F | negative | **positive** | negative | negative |
| BOB216 | 26 | C. 21-40 | F | negative | **positive** | negative | **positive** |
| BOB245 | 53 | D. >40 | M | **positive** | **positive** | negative | negative |
| BOB251 | 42 | D. >40 | M | negative | **positive** | negative | **positive** |
| BOB536 | 42 | D. >40 | F | negative | **positive** | negative | negative |
| BOB583 | 56 | D. >40 | F | **positive** | **positive** | negative | negative |
| BOB323 | 61 | D. >40 | M | negative | **positive** | negative | negative |
| BOB327 | 43 | D. >40 | M | negative | **positive** | negative | **positive** |
| BOB520 | 61 | D. >40 | M | **positive** | **positive** | negative | negative |
| DAN86 | 35 | C. 21-40 | F | negative | **positive** | negative | **positive** |
| KOK14 | 20 | B. 13-20 | M | negative | **positive** | negative | **positive** |
| ZAN2 | 13 | B. 13-20 | M | negative | **positive** | **positive** | **positive** |
| PAD63 | 27 | C. 21-40 | M | negative | **positive** | negative | **positive** |
| FO75 | 6 | A. 5-12 | M | negative | **positive** | **positive** | **positive** |
| BOB63 | 35 | C. 21-40 | M | **positive** | negative | negative | negative |
| BOB161 | 66 | D. >40 | M | **positive** | negative | negative | negative |
| BOB198 | 51 | D. >40 | F | **positive** | negative | negative | negative |
| BOB202 | 29 | C. 21-40 | F | **positive** | negative | negative | negative |
| BOB209 | 45 | D. >40 | F | **positive** | negative | negative | negative |
| BOB262 | 7 | A. 5-12 | M | **positive** | negative | **positive** | **positive** |
| BOB321 | 71 | D. >40 | M | **positive** | negative | negative | negative |
| BOB322 | 69 | D. >40 | F | **positive** | negative | negative | negative |
| BOB355 | 15 | B. 13-20 | F | **positive** | negative | negative | **positive** |
| BOB356 | 13 | B. 13-20 | F | **positive** | negative | negative | **positive** |
| BOB367 | 14 | B. 13-20 | F | **positive** | negative | **positive** | **positive** |
| BOB380 | 16 | B. 13-20 | F | **positive** | negative | negative | **positive** |
| BOB454 | 9 | A. 5-12 | M | **positive** | negative | negative | negative |
| BOB468 | 11 | A. 5-12 | M | **positive** | negative | **positive** | **positive** |
| BOB469 | 11 | A. 5-12 | M | **positive** | negative | **positive** | **positive** |
| BOB492 | 15 | B. 13-20 | F | **positive** | negative | negative | negative |
| BOB497 | 12 | A. 5-12 | M | **positive** | negative | negative | negative |
| BOB508 | 12 | A. 5-12 | M | **positive** | negative | negative | negative |
| BOB534 | 55 | D. >40 | F | **positive** | negative | negative | negative |
| BOB546 | 57 | D. >40 | F | **positive** | negative | **positive** | **positive** |
| BOB564 | 63 | D. >40 | F | **positive** | negative | **positive** | **positive** |
| BOB587 | 56 | D. >40 | F | **positive** | negative | negative | negative |
| BOB596 | 56 | D. >40 | M | **positive** | negative | negative | negative |
| DAN38 | 13 | B. 13-20 | M | **positive** | negative | **positive** | **positive** |
| DAN60 | 13 | B. 13-20 | F | **positive** | negative | **positive** | **positive** |

**Supplementary References**

1. Majiya H, Aliyu-Paiko M, Balogu VT, Musa DA, Salihu IM, Kawu AA, et al. Seroprevalence of SARS-CoV-2 in Niger State: A Pilot Cross Sectional Study. medRxiv [Internet]. 2021 Jun 20 [cited 2022 Jul 4];2020.08.04.20168112. Available from: https://www.medrxiv.org/content/10.1101/2020.08.04.20168112v2

2. Asuquo MI, Effa E, Otu A, Ita O, Udoh U, Umoh V, et al. Prevalence of IgG and IgM antibodies to SARS-CoV-2 among clinic staff and patients. medRxiv [Internet]. 2020 Jul 24 [cited 2022 Jul 6];2020.07.02.20145441. Available from: https://www.medrxiv.org/content/10.1101/2020.07.02.20145441v2

3. Ifeorah I, Nna E. Sero-pravelence of SARS CoV-2 IgM and IgG Antibodies Amongst Blood Donors in Nigeria. 2021 Jan 21 [cited 2022 Jul 5]; Available from: https://www.researchsquare.com

4. Olayanju O, Bamidele O, Edem F, Eseile B, Amoo A, Nwaokenye J, et al. SARS-CoV-2 Seropositivity in Asymptomatic Frontline Health Workers in Ibadan, Nigeria. Am J Trop Med Hyg [Internet]. 2021 Jan 6 [cited 2022 Jul 6];104(1):91–4. Available from: https://pubmed.ncbi.nlm.nih.gov/33185181/

5. Audu ID RA, Stafford ID KA, Steinhardt LI, Musa ID ZA, IriemenamID N, IloriID E, et al. Seroprevalence of SARS-CoV-2 in four states of Nigeria in October 2020: A population-based household survey. PLOS Glob Public Heal [Internet]. 2022 Jun 17;2(6). Available from: https://doi.org/10.1371/journal.pgph.0000363

6. Benn CS, Salinha A, Mendes S, Cabral C, Martins C, Nielsen S, et al. SARS-CoV-2 serosurvey among adults involved in healthcare and health research in Guinea-Bissau, West Africa. Public Health. 2022 Feb 1;203:19–22.

7. Talla C, Loucoubar C, Roka JL, Barry MA, Ndiaye S, Diarra M, et al. Seroprevalence of anti-SARS-CoV-2 antibodies in Senegal: a national population-based cross-sectional survey, between October and November 2020. IJID Reg. 2022 Jun 1;3:117–25.

8. Halatoko WA, Konu YR, Gbeasor-Komlanvi FA, Sadio AJ, Tchankoni MK, Komlanvi KS, et al. Prevalence of SARS-CoV-2 among high-risk populations in Lomé (Togo) in 2020. PLoS One [Internet]. 2020 Nov 1 [cited 2022 Jul 5];15(11):e0242124. Available from: https://journals.plos.org/plosone/article?id=10.1371/journal.pone.0242124

9. Milleliri JM, Coulibaly D, Nyobe B, Rey JL, Lamontagne F, Hocqueloux L, et al. SARS-CoV-2 Infection in Ivory Coast: A Serosurveillance Survey among Gold Mine Workers. Am J Trop Med Hyg [Internet]. 2021 May 5 [cited 2022 Jul 5];104(5):1709–12. Available from: https://pubmed.ncbi.nlm.nih.gov/33735104/

10. Barrie MB, Lakoh S, Kelly JD, Kanu JS, Squire JS, Koroma Z, et al. SARS-CoV-2 antibody prevalence in Sierra Leone, March 2021: a cross-sectional, nationally representative, age-stratified serosurvey. BMJ Glob Heal [Internet]. 2021;6:7271. Available from: http://gh.bmj.com/

11. Sagara I, Woodford J, Kone M, Assadou MH, Katile A, Attaher O, et al. Rapidly Increasing Severe Acute Respiratory Syndrome Coronavirus 2 Seroprevalence and Limited Clinical Disease in 3 Malian Communities: A Prospective Cohort Study. Clin Infect Dis [Internet]. 2022 Mar 15 [cited 2022 Sep 15];74(6):1030–8. Available from: https://pubmed.ncbi.nlm.nih.gov/34185847/

12. Wiens KE, Mawien PN, Rumunu J, Slater D, Jones FK, Moheed S, et al. Seroprevalence of severe acute respiratory syndrome coronavirus 2 IgG in Juba, South Sudan, 2020. Emerg Infect Dis. 2021;27(6):1598–606.

13. Kempen JH, Abashawl A, Suga HK, Difabachew MN, Kempen CJ, Debele MT, et al. SARS-CoV-2 serosurvey in Addis Ababa, Ethiopia. Am J Trop Med Hyg. 2020;103(5):2022–3.

14. Shaweno T, Abdulhamid I, Bezabih L, Teshome D, Derese B, Tafesse H, et al. Seroprevalence of SARS-CoV-2 antibody among individuals aged above 15 years and residing in congregate settings in Dire Dawa city administration, Ethiopia. Trop Med Health [Internet]. 2021 Dec 1 [cited 2022 Jul 6];49(1):1–8. Available from: https://tropmedhealth.biomedcentral.com/articles/10.1186/s41182-021-00347-7

15. Tadesse EB, Endris AA, Solomon H, Alayu M, Kebede A, Eshetu K, et al. Seroprevalence and risk factors for SARS-CoV-2 Infection in selected urban areas in Ethiopia: a cross-sectional evaluation during July 2020. Int J Infect Dis [Internet]. 2021;111:179–85. Available from: https://doi.org/10.1016/j.ijid.2021.08.028

16. Abdella S, Riou S, Tessema M, Assefa A, Seifu A, Blachman A, et al. Prevalence of SARS-CoV-2 in urban and rural Ethiopia: Randomized household serosurveys reveal level of spread during the first wave of the pandemic. EClinicalMedicine [Internet]. 2021 May 1 [cited 2022 Jul 5];35. Available from: https://pubmed.ncbi.nlm.nih.gov/34124630/

17. Nega Alemu B, Addissie A, Mamo G, Deyessa N, Abebe T, Abagero A, et al. Sero-prevalence of anti-SARS-CoV-2 Antibodies in Addis Ababa, Ethiopia. Ethiopia; 2021.

18. Uyoga S, Adetifa IMO, Karanja HK, Nyagwange J, Tuju J, Wanjiku P, et al. Seroprevalence of anti-SARS-CoV-2 IgG antibodies in Kenyan blood donors. Science (80- ). 2021;371(6524):79–82.

19. Ngere I, Dawa J, Hunsperger E, Otieno N, Masika M, Amoth P, et al. High seroprevalence of SARS-CoV-2 but low infection fatality ratio eight months after introduction in Nairobi, Kenya. Int J Infect Dis [Internet]. 2021 Nov 1 [cited 2022 Jul 4];112:25–34. Available from: https://pubmed.ncbi.nlm.nih.gov/34481966/

20. Chibwana MG, Jere KC, Kamng’ona R, Mandolo J, Katunga-Phiri V, Tembo D, et al. High SARS-CoV-2 seroprevalence in health care workers but relatively low numbers of deaths in urban Malawi. Wellcome Open Res 2020 5199 [Internet]. 2020 Dec 18 [cited 2022 Jul 5];5:199. Available from: https://wellcomeopenresearch.org/articles/5-199

21. Nwosu K, Fokam J, Wanda F, Mama L, Orel E, Ray N, et al. SARS-CoV-2 antibody seroprevalence and associated risk factors in an urban district in Cameroon. Nat Commun [Internet]. 2021 Dec 1 [cited 2022 Jul 4];12(1). Available from: /pmc/articles/PMC8494753/

22. Mveang Nzoghe A, Leboueny M, Kuissi Kamgaing E, Maloupazoa Siawaya AC, Bongho EC, Mvoundza Ndjindji O, et al. Circulating anti-SARS-CoV-2 nucleocapsid (N)-protein antibodies and anti-SARS-CoV-2 spike (S)-protein antibodies in an African setting: herd immunity, not there yet! BMC Res Notes [Internet]. 2021 Dec 1 [cited 2022 Jul 5];14(1):1–4. Available from: https://bmcresnotes.biomedcentral.com/articles/10.1186/s13104-021-05570-3

23. Batchi-Bouyou AL, Lobaloba Ingoba L, Ndounga M, Vouvoungui JC, Mfoutou Mapanguy CC, Boumpoutou KR, et al. High SARS-CoV-2 IgG/IGM seroprevalence in asymptomatic Congolese in Brazzaville, the Republic of Congo. Int J Infect Dis [Internet]. 2021 May 1 [cited 2022 Jul 5];106:3–7. Available from: https://pubmed.ncbi.nlm.nih.gov/33370565/

24. Mukwege D, Byabene AK, Akonkwa EM, Dahma H, Dauby N, Buhendwa JPC, et al. High SARS-CoV-2 Seroprevalence in Healthcare Workers in Bukavu, Eastern Democratic Republic of Congo. Am J Trop Med Hyg [Internet]. 2021 Apr 1 [cited 2022 Jul 5];104(4):1526–30. Available from: https://pubmed.ncbi.nlm.nih.gov/33591936/

25. Kammon AM, El-Arabi AA, Erhouma EA, Mehemed TM, Mohamed OA. Seroprevalence of antibodies against SARS-CoV-2 among public community and health-care workers in Alzintan City of Libya. medRxiv [Internet]. 2020 May 26 [cited 2022 Jul 5];2020.05.25.20109470. Available from: https://www.medrxiv.org/content/10.1101/2020.05.25.20109470v1

26. Gomaa MR, El Rifay AS, Shehata M, Kandeil A, Nabil Kamel M, Marouf MA, et al. Incidence, household transmission, and neutralizing antibody seroprevalence of Coronavirus Disease 2019 in Egypt: Results of a community-based cohort. PLoS Pathog [Internet]. 2021 Mar 1 [cited 2022 Jul 5];17(3):e1009413. Available from: https://pubmed.ncbi.nlm.nih.gov/33705496/

27. Mulenga LB, Hines JZ, Fwoloshi S, Chirwa L, Siwingwa M, Yingst S, et al. Prevalence of SARS-CoV-2 in six districts in Zambia in July, 2020: a cross-sectional cluster sample survey. Lancet Glob Heal [Internet]. 2021;9(6):e773–81. Available from: http://dx.doi.org/10.1016/S2214-109X(21)00053-X

28. Hines JZ, Fwoloshi S, Kampamba D, Barradas DT, Banda D, Zulu JE, et al. SARS-CoV-2 Prevalence among Outpatients during Community Transmission, Zambia, July 2020 - Volume 27, Number 8—August 2021 - Emerging Infectious Diseases journal - CDC. Emerg Infect Dis [Internet]. 2021 Aug 1 [cited 2022 Jul 6];27(8):2166–8. Available from: https://wwwnc.cdc.gov/eid/article/27/8/21-0502_article

29. Shaw JA, Meiring M, Cummins T, Chegou NN, Claassen C, Du Plessis N, et al. Higher SARS-CoV-2 seroprevalence in workers with lower socioeconomic status in Cape Town, South Africa. PLoS One [Internet]. 2021 Feb 1 [cited 2022 Sep 19];16(2). Available from: https://pubmed.ncbi.nlm.nih.gov/33630977/

30. Sykes W, Mhlanga L, Swanevelder R, Glatt TN, Grebe E, Coleman C, et al. Prevalence of anti-SARS-CoV-2 antibodies among blood donors in Northern Cape, KwaZulu-Natal, Eastern Cape, and Free State provinces of South Africa in January 2021. Res Sq [Internet]. 2021 Feb 12 [cited 2022 Jul 6];7. Available from: /pmc/articles/PMC7885925/

31. Wolter N, Tempia S, Von Gottberg A, Bhiman JN, Walaza S, Kleynhans J, et al. Seroprevalence of SARS-CoV-2 after the Second Wave in South Africa in HIV-Infected and Uninfected Persons: A Cross-Sectional Household Survey, November 2020 – April 2021. SSRN Electron J [Internet]. 2021 Nov 6 [cited 2022 Jul 6]; Available from: https://papers.ssrn.com/abstract=3957112

32. Vermeulen M, Mhlanga L, Sykes W, Coleman C, Pietersen N, Cable R, et al. Prevalence of anti-SARS-CoV-2 antibodies among blood donors in South Africa during the period. Res Sq [Internet]. 2021 [cited 2022 Jul 6]; Available from: https://doi.org/10.21203/rs.3.rs-690372/v2

33. Amoo OS, Aina OO, Okwuraiwe AP, Onwuamah CK, Shaibu JO, Ige F, et al. COVID-19 Spread Patterns Is Unrelated to Malaria Co-Infections in Lagos, Nigeria. Adv Infect Dis. 2020;10(05):200–15.

34. Muhammad Y, Aminu YK, Ahmad AE, Iliya S, Muhd N, Yahaya M, et al. An elevated 8-isoprostaglandin F2 alpha (8-iso-PGF2α) in COVID-19 subjects co-infected with malaria. Pan Afr Med J [Internet]. 2020 Sep 21 [cited 2022 Sep 15];37(78):1–10. Available from: /pmc/articles/PMC7680236/

35. Onosakponome EO, Wogu MN. The Role of Sex in Malaria-COVID19 Coinfection and Some Associated Factors in Rivers State, Nigeria. J Parasitol Res. 2020;2020.

36. Matangila JR, Nyembu RK, Telo GM, Ngoy CD, Sakobo TM, Massolo JM, et al. Clinical characteristics of COVID-19 patients hospitalized at Clinique Ngaliema, a public hospital in Kinshasa, in the Democratic Republic of Congo: A retrospective cohort study. PLoS One [Internet]. 2020;15(12 December):1–15. Available from: http://dx.doi.org/10.1371/journal.pone.0244272

37. Achan J, Serwanga A, Wanzira H, Kyagulanyi T, Nuwa A, Magumba G, et al. Current malaria infection, previous malaria exposure, and clinical profiles and outcomes of COVID-19 in a setting of high malaria transmission: an exploratory cohort study in Uganda. The Lancet Microbe [Internet]. 2022;3(1):e62–71. Available from: http://dx.doi.org/10.1016/S2666-5247(21)00240-8

38. Bakamutumaho B, Cummings MJ, Owor N, Kayiwa J, Namulondo J, Byaruhanga T, et al. Severe COVID-19 in uganda across two epidemic phases: A prospective cohort study. Am J Trop Med Hyg. 2021;105(3):740–4.

39. Morton B, Barnes KG, Anscombe C, Jere K, Matambo P, Mandolo J, et al. Distinct clinical and immunological profiles of patients with evidence of SARS-CoV-2 infection in sub-Saharan Africa. Nat Commun. 2021;12(1).

40. Mahajan NN, Gajbhiye RK, Bahirat S, Lokhande PD, Mathe A, Rathi S, et al. Co-infection of malaria and early clearance of SARS-CoV-2 in healthcare workers. J Med Virol. 2021;93(4):2431–8.
